# Mesenteric lymphadenopathy is a key to diagnosis of radiologically challenging pulmonary lymphoma

**DOI:** 10.1111/1759-7714.13453

**Published:** 2020-04-29

**Authors:** Pei‐Hsuan Lu, Chung‐Yao Huang, Zong‐Yi Jhou, Wei‐Ming Huang, Chia‐Hung Chen, Chun‐Chao Huang

**Affiliations:** ^1^ Department of Radiology MacKay Memorial Hospital Taipei Taiwan; ^2^ Mackay Junior College of Medicine Nursing, and Management Taipei Taiwan; ^3^ Department of Medicine MacKay Medical College New Taipei City Taiwan

**Keywords:** Extranodal marginal zone B‐cell lymphoma, lung tumors, mesenteric lymphadenopathy, mucosa‐associated lymphoid tissue lymphoma, pulmonary lymphoma

## Abstract

Extranodal marginal zone B‐cell lymphoma (EMZBL), previously known as mucosa‐associated lymphoid tissue lymphoma, is the most common type of marginal zone B‐cell lymphomas. Primary pulmonary lymphomas only constitute 0.5% of primary lung cancer, but 90% of these are EMZBLs. Primary pulmonary lymphomas share similar imaging features with secondary pulmonary lymphomas. Imaging diagnosis is challenging because many benign and other malignant lung lesions can display similar features. Here, we demonstrate a 70‐year‐old male case with lung tumors and only mesenteric lymphadenopathy, which was eventually diagnosed as advanced pulmonary EMZBL with involvement of the mesenteric lymph nodes and bone marrow. Pulmonary masses have a wide differential diagnosis, but concurrent isolated mesenteric lymphadenopathy might be a radiological clue to pulmonary lymphoma.

**Key points:**

1. Concurrent isolated mesenteric lymphadenopathy might be a radiological clue to pulmonary lymphoma.
2. For nonspecific lung tumors, additional abdominal computed tomography (CT) scan might be helpful for diagnosis of possible lymphoma.

## Introduction

Extranodal marginal zone B‐cell lymphoma (EMZBL), previously known as mucosa‐associated lymphoid tissue (MALT) lymphoma, is the most common type (50%–70%) of marginal zone B‐cell lymphomas, which account for 5%–17% of all non‐Hodgkin’s lymphomas.^1^, ^2^ Primary pulmonary lymphomas only constitute 0.5% of primary lung cancer but 90% of these are EMZBLs.^3^ Pulmonary EMZBLs share similar imaging features with secondary pulmonary lymphomas and these features include masses, consolidation, nodules, air bronchogram, angiogram sign, and halo sign with ground‐glass shadowing.^4^, ^5^ Although less common, lymphadenopathy is reported up to 29% in pulmonary EMZBLs.^4^ In advanced cases, bone marrow involvement can occur and is found in 13%–30% of cases.^6^ Mesenteric lymphadenopathy in non‐Hodgkin’s lymphoma is about 45% with only 6% extrapulmonary nodal involvement, including abdominal lymphadenopathy, reported in pulmonary EMZBL, which is a subtype of non‐Hodgkin’s lymphoma.^7^, ^8^ Imaging diagnosis is challenging because many benign and other malignant lung lesions can display similar features.^4^ Here, we demonstrate a case with lung tumors and only mesenteric lymphadenopathy, which is a rare finding in primary pulmonary infectious processes or primary lung carcinomas, but can be an important diagnostic clue to lymphoma.

### Case report

**Figure 1:**
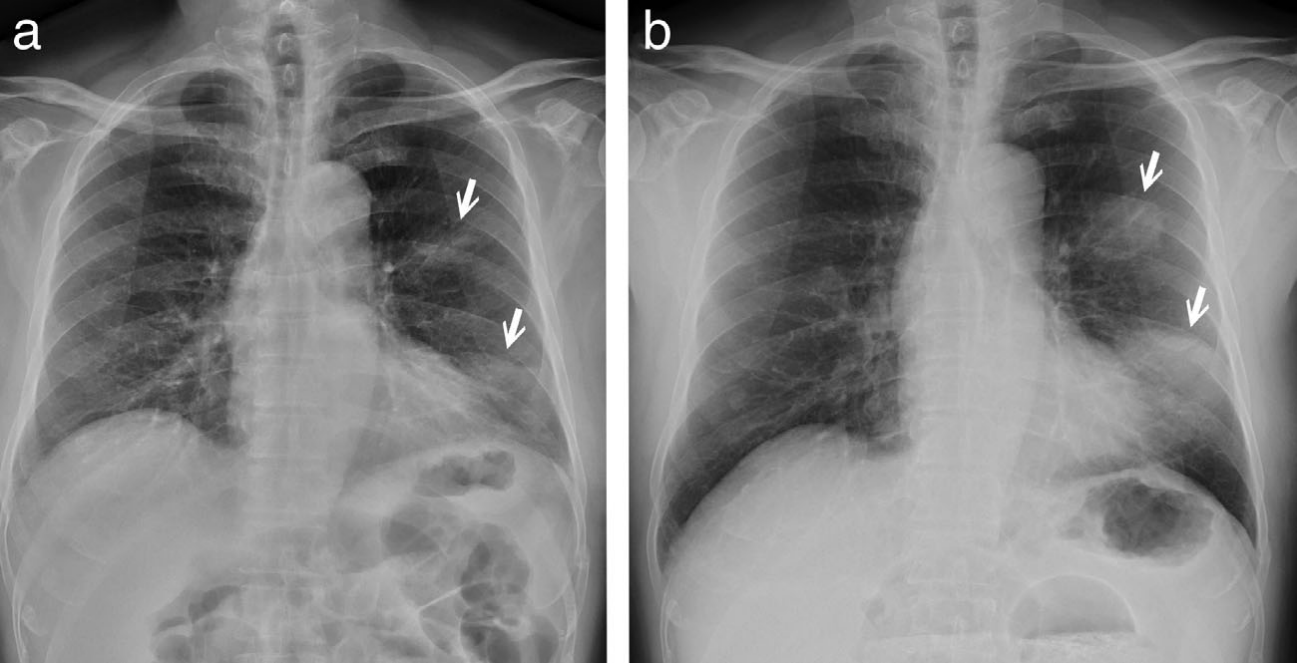
(**a**) Chest radiograph showed ill‐defined opacities in the left parahilar and left lower lung fields (arrows in a). (**b**) Follow‐up chest radiograph three months later revealed a rapidly developed well‐defined nodular and consolidated lesions in the left middle and lower lung fields (arrows in b).

**Figure 2:**
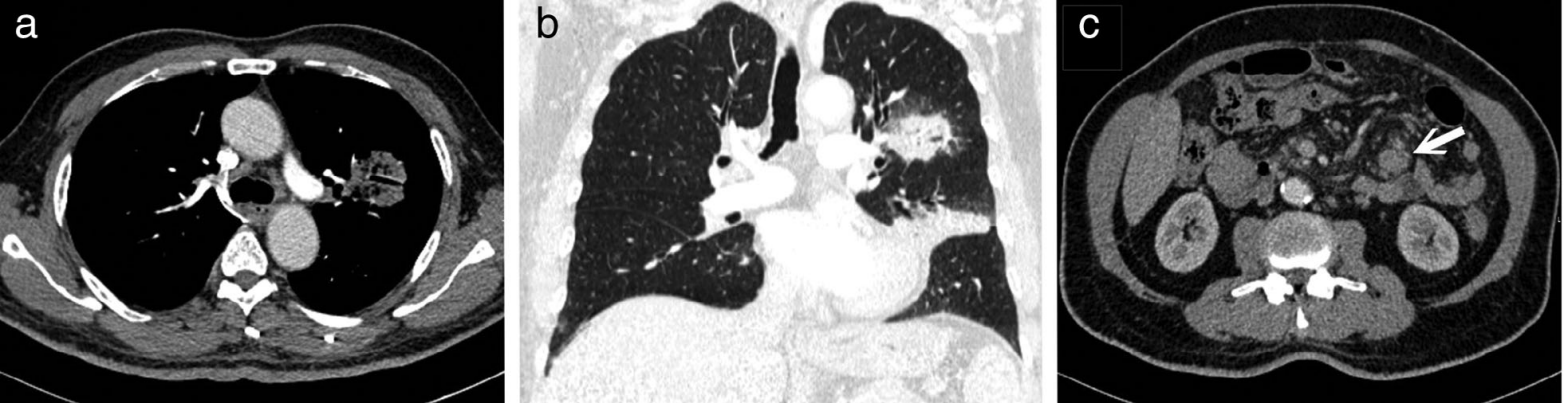
(**a**) Computed tomography (CT) scan of the chest demonstrated a 3.9 cm consolidative mass with air bronchogram in the left suprahilar region. (**b**) Lung window of a reformatted coronal plane image revealed ground‐glass opacities at the periphery of the left suprahilar mass and consolidation in the left inferior lingula. (**c**) The visible upper abdomen in the same CT scan showed a mildly enlarged mesenteric LN (arrow).

A 70‐year‐old male patient was seen at our chest outpatient department with symptoms of a chronic productive cough without fever for months. He had type 2 diabetes mellitus, hyperlipidemia and chronic ischemic heart disease. He was a cement industry owner and an ex‐smoker (half pack per day for 50 years). When comparing the chest radiograph which had been taken three months previously, the ill‐defined opacities that had been visible at that time in the left parahilar and lower lung fields had rapidly progressed to nodular and consolidated lesions (Fig 1a,b). Physical examination and laboratory tests were unremarkable. Computed tomography (CT) scan of the chest showed a large consolidative lesion with air bronchogram in the left inferior lingula; a 3.9 cm consolidative mass with air bronchogram, ground glass margin in the left suprahilar region (Fig 2a,b) and another smaller one in the anterior segment of right upper lobe (RUL). In addition, some subcentimeter or mildly enlarged lymph nodes (LNs) in the mesentery (Fig 2c) were seen. A multifocal infectious process or pneumonic type lung neoplasm was initially suspected. Transbronchial biopsy was performed and the pathologic report showed atypical lymphoid infiltrate. Additional immunohistochemical study revealed that the CD20 and Bcl‐2 stains were positive, supporting low‐grade B cell lymphoma. CD3, CD5, cyclin D1, Bcl‐6 and CD23 stains excluded mantle cell lymphoma, small lymphocytic lymphoma and follicular lymphoma. Molecular study of IgH gene clonality polymerase chain reaction (PCR) analysis showed presence of oligoclonal rearranged IgH gene, proving clonal B cell proliferation. Further, F‐18 fluorodeoxyglucose positron emission tomography (F‐18 FDG PET) scan showed large hypermetabolic lesions in the left middle lung field, and another smaller focus in the right middle lung field without abnormal radiotracer uptake in the abdomen. Left iliac bone marrow biopsy revealed hypercellular marrow with trilineage hyperplasia and low grade B cell lymphoma involvement. It was concluded that the patient had EMZBL of the lung, stage IVA with bone marrow involvement. The patient commenced chemotherapy treatment with COP (cyclophosphamide, vincristine and methylprednisone) regimen. The patient had been followed for one and half year and he was doing well with partial shrinkage of the lung lesions and mesenteric lymphadenopathy after treatment.

## Discussion

MALT lymphoma, also known as a low grade EMZBL, is usually seen in the gastrointestinal tract, salivary gland and other organs. It rarely has pulmonary involvement but is the most common subset of primary pulmonary lymphoma representing 90% of cases.^9^

The common imaging characteristics of pulmonary MALT lymphoma include consolidation, pulmonary masses or nodules with multiple, bilateral involvement and random distribution.^4^, ^10^ Associated findings include air bronchograms, angiogram signs or a halo of ground‐glass opacity at the lesion margin.^4^ Although mesenteric lymphadenopathy is often found in non‐Hodgkin’s lymphoma, the overall extrapulmonary nodal involvement is only 6% in pulmonary EMZBL.^7^, ^8^ In the present case, the chest CT scan showed a mass‐like lesion with a halo of marginal ground‐glass opacity in the left suprahilar area, another similar but smaller one in the anterior segment of right upper lobe, and one consolidative lesion with air bronchogram in the left inferior lingula. All these findings can be imaging features of pulmonary EMZBL. In addition, based on previous imaging, rapidly developed lesions in the left lung field and some enlarged abdominal LNs may indicate pulmonary lymphoma rather than primary lung carcinomas or infectious processes and the patient given an appropriate treatment strategy. Primary lung carcinomas with isolated abdominal lymphadenopathy are reported extremely rarely.^11^ Pneumonia with mesenteric lymphadenopathy is also rare and usually occurs in children with abdominal pain.^12^

In conclusion, differential diagnosis varies for pulmonary masses but concurrent isolated mesenteric lymphadenopathy might be a radiological clue to pulmonary lymphoma.

## Disclosure

The authors declare there is no conflict of interest.
